# Editorial: The Role of the Renin-Angiotensin System in the Central Nervous System

**DOI:** 10.3389/fnins.2021.733084

**Published:** 2021-07-28

**Authors:** Natalia P. Rocha, Ana Cristina Simões e Silva, Antonio Lucio Teixeira

**Affiliations:** ^1^Department of Neurology, The Mitchell Center for Alzheimer's Disease and Related Brain Disorders, McGovern Medical School, The University of Texas Health Science Center at Houston, Houston, TX, United States; ^2^Laboratório Interdisciplinar de Investigação Médica, Faculdade de Medicina, Universidade Federal de Minas Gerais (UFMG), Belo Horizonte, Brazil; ^3^Neuropsychiatry Program, Department of Psychiatry and Behavioral Sciences, McGovern Medical School, The University of Texas Health Science Center at Houston, Houston, TX, United States

**Keywords:** renin-angiotensin system, angiotensin, angiotensin-converting enzyme, brain, neuropsychiatry

The Renin-Angiotensin System (RAS) has been known for decades as a system involved in blood pressure control through the regulation of volemia and systemic vascular resistance. More importantly, angiotensin-converting enzyme (ACE) inhibitors and angiotensin (Ang) II receptor antagonists have considerably contributed to decreasing morbidity and mortality due to hypertension (Basso and Terragno, 2001), bringing the RAS to the front light of cardiovascular medicine.

The classical vasopressor effects of the RAS involve a series of physiological pathways initiated by renin, an enzyme secreted by the kidneys. Renin hydrolyzes angiotensinogen to produce Ang I which, in turn, is cleaved by the ACE into Ang II. The main effects of the RAS are thought to be exerted by Ang II through the angiotensin type 1 (AT_1_) receptor. ACE, Ang II, and AT_1_ receptor constitute the main players of the classical arm of RAS, whose effects include vasoconstriction, aldosterone secretion, and fluid retention.

Our knowledge about the RAS has grown exponentially over the years as a multitude of other enzymes, peptides, and receptors have been identified. In this context, it is worth highlighting Ang-(1–7), a heptapeptide produced mainly by the cleavage of Ang II by an enzyme homolog to ACE, the ACE2 (Donoghue et al., 2000; Tipnis et al., 2000). Most physiological effects of Ang-(1–7) through its receptor “Mas” antagonize the classical RAS actions. Therefore, the RAS arm composed of ACE2/Ang-(1–7)/Mas receptor is regarded as a counter-regulatory axis of the classical arm of the RAS (Santos et al., 2008).

Our understanding of the physiological roles of the RAS has also evolved based on the findings of RAS components in “unlikely” places—for example, renin (a kidney enzyme) in the brain. As a result, the concept of local RAS was introduced (Paul et al., 2006), and herein we highlight the brain RAS. At first, the physiological effects linked to the brain RAS were associated with cardiovascular and body fluid homeostasis, such as the control of drinking behavior, salt appetite, vasopressin release, baroreflex, and sympathetic/parasympathetic activity. Later, it became clear that RAS actions in the central nervous system (CNS) are not limited to blood pressure and water-electrolyte balance regulation. Actually, the RAS is involved in several brain functions, including cognition, motor, and emotional/behavioral modulation (Rocha et al., 2018a). In this regard, the RAS has been implicated in the pathophysiology or related to the clinical phenotype observed in different neuropsychiatric conditions, such as Alzheimer' disease (AD) (Rocha et al., 2018b), Parkinson's disease (Rocha et al., 2016), schizophrenia (Mohite et al., 2018), and mood disorders (Mohite et al., 2020).

The manuscripts published in the current Research Topic discuss the role played by the RAS in the CNS and in CNS-related diseases. Regarding the traditional role attributed to the RAS (i.e., blood pressure control), Xue et al. revisited the evidence on how the RAS and immune mechanisms in the CNS synergistically induce a hypertensive response sensitization contributing to the pathogenesis of systemic hypertension. For example, stressors such as low-dose of Ang II and high-fat diet can induce the upregulation of both RAS and inflammatory elements, which will act on brain nuclei involved in blood pressure regulation through systemic-brain communication (Xue et al.). A second review discussed the interactions between peripheral- and CNS- RAS in the pathophysiology of systemic hypertension. The authors emphasized the relevance of the bidirectional crosstalk between the bone marrow RAS and the CNS RAS in atherosclerosis and hypertension. They also presented potential pharmacological approaches for treating cardiovascular diseases by activating the ACE2/Ang-(1–7)/Mas receptor pathway (Ciftciler and Haznedaroglu). Focusing on the CNS-related mechanisms underlying the antihypertensive effects mediated by Ang-(1–7), the original study performed by Kangussu et al. demonstrated that chronic intracerebroventricular infusion of Ang-(1–7) modulates both inflammatory and RAS-related responses in the hypothalamus. More specifically, i.c.v. administration of Ang-(1–7) resulted in changes in inflammatory (TNF decrease and IL-10 increase) and RAS-related components (reduced AT_1_ receptor expression and ACE activity) in the hypothalamus, besides reducing cardiac hypertrophy in hypertensive transgenic (mRen2)27 rats (Kangussu et al.).

Hypertension is a common comorbidity and also a risk factor for neuropsychiatric diseases. Accordingly, the RAS may be regarded as a common pathophysiological pathway shared by these conditions. For example, hypertension is commonly observed in individuals with epilepsy, while lower blood pressure has been associated with reduced frequency and severity of seizures in patients with epilepsy. Becari et al. investigated whether the RAS is involved in seizures evoked by chronic acoustic stimulation in spontaneously hypertensive rats (SHRs). The chronic treatment with RAS-acting agents, such as ACE inhibitors and AT_1_ receptor antagonists, reduced blood pressure in SHR but did not influence seizures. The authors concluded that the RAS might not play a major role in the mechanisms underlying audiogenic seizures in SHR (Becari et al.).

In addition to the role played by the RAS in CNS control of blood pressure and epilepsy, recent studies have investigated the involvement of the RAS in neurodegenerative diseases. The rationale behind these studies relies on the connection between RAS and different mechanisms implicated in neurodegeneration, including oxidative stress, neuroinflammation, endothelial dysfunction, microglial polarization, and alterations in neurotransmitter secretion, as reviewed by Cosarderelioglu et al. An exploratory study assessed whether peripheral levels of RAS components are associated with structural brain measures in patients with AD. Patients with AD presented reduced plasma levels of Ang-(1–7) in comparison with age-matched controls. Among patients with AD, lower levels of Ang-(1–7) were associated with decreased white matter hypointensities volumes. These findings corroborate the hypothesis that the RAS counter-regulatory/neuroprotective arm is downregulated in AD [here evidenced by lower levels of Ang-(1–7)], and possibly associated with cerebrovascular lesions in AD (Ribeiro et al.). Peripheral levels of RAS components were also investigated in relation to clinical outcomes (specifically cognitive performance) in patients with Huntington's disease (HD). In the study performed by Rocha et al., higher levels of ACE2 were associated with better scores in the Verbal Fluency Test, while higher levels of Ang II correlated with worse scores on the Mini-Mental State Examination in patients with HD. These results corroborate the proposed hypothesis of an imbalance between the classical (ACE/Ang II/AT_1_ receptor) and the counter-regulatory/neuroprotective [ACE2/Ang-(1–7)/Mas receptor] arms of the RAS, being the former associated with negative clinical outcomes and the latter with positive effects in HD (Rocha et al.). Given the aforementioned results, it is tempting to hypothesize that the ACE/Ang II/AT_1_ receptor axis inhibition and/or ACE2/Ang-(1–7)/Mas receptor axis activation would result in neuroprotective effects and/or positive clinical outcomes. With this perspective in mind, the treatment with candesartan (an AT_1_ antagonist with documented ability to cross the blood-brain barrier) was tested as neuroprotective in a preclinical model of traumatic brain injury and showed to improve functional recovery and reduce neuropathology. Attilio et al. investigated, on this Research Topic, through RNA sequence analysis the mechanisms by which candesartan treatment influences the response to injury. Candesartan induced changes in genes involved in angiogenesis, inflammatory response, and extracellular matrix regulation (Attilio et al.). This study confirmed that AT_1_ receptor antagonists are neuroprotective possibly due to a combination of effects, including anti-inflammatory properties. It is worth mentioning that the use of AT_1_ receptor antagonists not only blocks the Ang II/AT_1_ receptor signaling but also upregulates the Ang-(1–7)/Mas receptor pathway, thus the neuroprotective effects of such drugs are possibly due to the ACE2/Ang-(1–7)/Mas receptor axis activation as well (Rocha et al., 2018a).

Lastly, the role of the RAS in the CNS was investigated in the context of psychiatric disorders. Bipolar disorder (BD) is one of the most severe mental illnesses, with high rates of medical comorbidities and functional impairment. The etiopathogenesis of BD is yet to be elucidated and among the several pathways that might be involved, Sanches et al. hypothesized that dysfunctions in the RAS might play a role in the pathophysiology of this complex disorder. They reported no differences between patients with BD and age-matched controls regarding the plasma levels of ACE, ACE2, Ang-(1–7), and Ang II. However, non-euthymic patients with BD (i.e. patients experiencing mood episodes) had significantly lower ACE levels compared to controls. This study reinforces previous findings that RAS-related changes are associated with clinical outcomes in neuropsychiatric conditions (Sanches et al.). Given the medical and societal burden associated with psychiatric disorders and the associated therapeutic challenges, investigating the role of RAS in this context might open new venues for understanding the biology of these disorders and, ultimately, novel pharmacological strategies.

In conclusion, this Research Topic highlights the emerging role of RAS in the CNS under physiological and pathophysiological conditions. Despite several gaps and controversies in the field, the investigation of RAS mechanisms in the CNS seems a promising endeavor. Future studies may shed light on pathophysiological processes underlying neuropsychiatric diseases and novel therapeutic targets for these devastating conditions.

## Author Contributions

NR wrote the first draft of the manuscript. ACSS and AT wrote sections of the manuscript. All authors contributed to manuscript revision, read, and approved the submitted version.

## Conflict of Interest

The authors declare that the research was conducted in the absence of any commercial or financial relationships that could be construed as a potential conflict of interest.

## Publisher's Note

All claims expressed in this article are solely those of the authors and do not necessarily represent those of their affiliated organizations, or those of the publisher, the editors and the reviewers. Any product that may be evaluated in this article, or claim that may be made by its manufacturer, is not guaranteed or endorsed by the publisher.
